# Supplementary material for: Hygienic behaviors during the COVID-19 pandemic may decrease immunoglobulin G levels: Implications for Kawasaki disease
Source: PLoS One. 2022 Sep 28;17(9):e0275295. doi: 10.1371/journal.pone.0275295 (PMC9518924; doi:10.1371/journal.pone.0275295)
Supplement: S1 Table — (DOCX) [file pone.0275295.s006.docx]

**S1 Table.** Linear regression coefficients to explain normalized IgG in different age groups.

| Period | < 0.3 years | | 0.3 - 5 years | | ≥ 5 years | |
| --- | --- | --- | --- | --- | --- | --- |
| 1. Pre-COVID  (2010-2019) | n=6716 |  | n=2256 |  | n=7152 |  |
| 1.1 Univariate | Coefficient | P | Coefficient | P | Coefficient | P |
| Time* (years) | -0.0349 | P=0.0142 | -0.0949 | P=0.0002 | -0.121 | P<0.0001 |
| Adjusted R^2^ | 0.0007 | P=0.0142 | 0.0055 | P=0.0002 | 0.0083 | P<0.0001 |
| 1.2. Multivariate | Coefficient | P | Coefficient | P | Coefficient | P |
| Time (years) | -0.00153 | P=0.9035 | -0.0853 | P=0.0002 | -0.0999 | P<0.0001 |
| Age† (years) | -33.1 | P<0.0001 | 1.09 | P<0.0001 | 0.0355 | P<0.0001 |
| Adjusted R^2^ | 0.2133 | P<0.0001 | 0.2011 | P<0.0001 | 0.0698 | P<0.0001 |
|  |  |  |  |  |  |  |
| 2. COVID  (2020-2021) | n=766 |  | n=577 |  | n=2277 |  |
| 1.1 Univariate | Coefficient | P | Coefficient | P | Coefficient | P |
| Time (years) | -0.912 | P<0.0001 | 0.0959 | P=0.6613 | -0.343 | P=0.0065 |
| Adjusted R^2^ | 0.0305 | P<0.0001 | -0.0014 | P=0.6613 | 0.0028 | P=0.0065 |
| 1.2. Multivariate | Coefficient | P | Coefficient | P | Coefficient | P |
| Time (years) | -0.421 | P=0.0039 | 0.204 | P=0.2782 | -0.339 | P=0.0052 |
| Age (years) | -30.5 | P<0.0001 | 1.21 | P<0.0001 | 0.0375 | P<0.0001 |
| Adjusted R^2^ | 0.367 | P<0.0001 | 0.2644 | P<0.0001 | 0.0799 | P<0.0001 |
