# Supplementary material for: Hygienic behaviors during the COVID-19 pandemic may decrease immunoglobulin G levels: Implications for Kawasaki disease
Source: PLoS One. 2022 Sep 28;17(9):e0275295. doi: 10.1371/journal.pone.0275295 (PMC9518924; doi:10.1371/journal.pone.0275295)
Supplement: S2 Table — (DOCX) [file pone.0275295.s007.docx]

**S2 Table.** Linear regression coefficients to explain IgG in four age groups

| Period | < 0.3 years | | 0.3 – 5 years | | 5 – 50 years | | ≥ 50 years | |
| --- | --- | --- | --- | --- | --- | --- | --- | --- |
| 1. Pre-COVID  (2010–2019) | n=6716 |  | n=2256 |  | n=3117 |  | n=4035 |  |
| 1.1 Univariate | Coefficient | P | Coefficient | P | Coefficient | P | Coefficient | P |
| Time* (years) | - 4.81 | P=0.0002 | - 10.0 | P<0.0001 | - 16.0 | P<0.0001 | - 13.0 | P=0.0002 |
| Adjusted R^2^ | 0.1843 | P=0.0002 | 0.0077 | P<0.0001 | 0.0119 | P<0.0001 | 0.0031 | P=0.0002 |
| 1.2. Multivariate | Coefficient | P | Coefficient | P | Coefficient | P | Coefficient | P |
| Time (years) | - 2.01 | P=0.0895 | - 9.2 | P<0.0001 | - 13.5 | P<0.0001 | - 13.4 | P=0.0001 |
| Age† (years) | - 2770 | P<0.0001 | 94 | P<0.0001 | 5.38 | P<0.0001 | 4.08 | P<0.0001 |
| Adjusted R^2^ | 0.1784 | P<0.0001 | 0.1841 | P<0.0001 | 0.0563 | P<0.0001 | 0.0080 | P<0.0001 |
|  |  |  |  |  |  |  |  |  |
| 2. COVID  (2020–2021) | n=766 |  | n=577 |  | n=1325 |  | n=952 |  |
| 2.1 Univariate | Coefficient | P | Coefficient | P | Coefficient | P | Coefficient | P |
| Time (years) | -82.1 | P<0.0001 | 4.23 | P=0.8261 | - 37.4 | P=0.0286 | - 39.2 | P=0.1847 |
| Adjusted R^2^ | 0.0305 | P<0.0001 | -0.0017 | P=0.8261 | 0.0029 | P=0.0286 | 0.0066 | P=0.1847 |
| 2.2. Multivariate | Coefficient | P | Coefficient | P | Coefficient | P | Coefficient | P |
| Time (years) | - 42.6 | P=0.0029 | 13.1 | P=0.4395 | - 42.1 | P=0.0139 | - 42.5 | P=0.1521 |
| Age (years) | - 2455 | P<0.0001 | 99.4 | P<0.0001 | 2.00 | P=0.0048 | 3.81 | P=0.0109 |
| Adjusted R^2^ | 0.3096 | P<0.0001 | 0.2292 | P<0.0001 | 0.0081 | P=0.0017 | 0.0066 | P=0.0162 |

*Time that elapsed from the beginning of study period to the date of IgG measurement (in years). † Age at the date of IgG measurement. For both time and age, the minimal temporal resolution used was day, while the unit of time is expressed in year.
