# Supplementary material for: Hygienic behaviors during the COVID-19 pandemic may decrease immunoglobulin G levels: Implications for Kawasaki disease
Source: PLoS One. 2022 Sep 28;17(9):e0275295. doi: 10.1371/journal.pone.0275295 (PMC9518924; doi:10.1371/journal.pone.0275295)
Supplement: S3 Table — (DOCX) [file pone.0275295.s008.docx]

**S3 Table.** Regression analysis to explain normalized IgE in different age groups

|  | < 5 years | | 5 - 20 years | | ≥ 20 years | |
| --- | --- | --- | --- | --- | --- | --- |
| 1. Pre-COVID  (2010-2019) | n=1990 |  | n=912 |  | n=1553 |  |
| 1. Univariate | Coefficient | P | Coefficient | P | Coefficient | P |
| Time (year) | 0.0235 | P=0.0740 | -0.00519 | P=0.7526 | -0.00234 | P=0.8585 |
| Adjusted R^2^ | 0.00111 | P=0.0740 | -0.0010 | P=0.7526 | -0.0006 | P=0.8585 |
| 1.2 Multivariate | Coefficient | P | Coefficient | P | Coefficient | P |
| Time (year) | 0.0124 | P=0.2949 | -0.0120 | P=0.4616 | -0.000141 | P=0.9914 |
| Age (year) | 0.494 | P<0.0001 | 0.0265 | P=0.0365 | -0.00548 | P=0.0097 |
| sin($2\pi$×Time) | -0.240 | P<0.0001 | -0.341 | P<0.0001 | -0.104 | P=0.0600 |
| cos($2\pi$×Time) | -0.0724 | P=0.0909 | -0.00435 | P=0.9492 | -0.0690 | P=0.2333 |
| Adjusted R^2^ | 0.2017 | P<0.0001 | 0.0303 | P<0.0001 | 0.0046 | 0.0253 |
|  |  |  |  |  |  |  |
| 2. COVID  (2020-2021) | n=245 |  | n=169 |  | n=564 |  |
| 2.1 Univariate | Coefficient | P | Coefficient | P | Coefficient | P |
| Time | 0.303 | P=0.0606 | 0.0987 | P=0.6391 | -0.331 | P=0.0031 |
| Adjusted R^2^ | 0.0104 | P=0.0606 | -0.0047 | P=0.6391 | 0.0137 | P=0.0031 |
| 2.2 Multivariate | Coefficient | P | Coefficient | P | Coefficient | P |
| Time (year) | 0.0812 | P=0.5859 | -0.0938 | P=0.6773 | -0.307 | P=0.0088 |
| Age (year) | 0.474 | P<0.0001 | 0.0778 | P=0.0136 | 0.00106 | P=0.7387 |
| sin($2\pi$×Time) | -0.375 | P=0.0011 | -0.317 | P=0.-740 | 0.0596 | P=0.5184 |
| cos($2\pi$×Time) | -0.186 | P=0.1001 | 0.189 | P=0.2645 | -0.0760 | P=0.3814 |
| Adjusted R^2^ | 0.2290 | P<0.0001 | 0.0358 | P=0.0406 | 0.0108 | P=0.0394 |
