# Supplementary material for: Hygienic behaviors during the COVID-19 pandemic may decrease immunoglobulin G levels: Implications for Kawasaki disease
Source: PLoS One. 2022 Sep 28;17(9):e0275295. doi: 10.1371/journal.pone.0275295 (PMC9518924; doi:10.1371/journal.pone.0275295)
Supplement: S4 Table — (DOCX) [file pone.0275295.s009.docx]

**S4 Table.** Regression coefficients which explain IgG, after excluding Kawasaki Disease cases.

| Period | < 0.3 years | | 0.3 – 5 years | | ≥ 5 years | |
| --- | --- | --- | --- | --- | --- | --- |
| 1. Pre-COVID  (2010–2019) | n=6,650 |  | n=1,905 |  | n=7,087 |  |
| 1.1 Univariate | Coefficient | P | Coefficient | P | Coefficient | P |
| Time* (years) | -4.65 | 0.0004 | -9.12 | 0.0004 | -16.4 | P<0.0001 |
| Adjusted R^2^ | 0.0017 | 0.0004 | 0.0061 | 0.0004 | 0.0067 | P<0.0001 |
| 1.2. Multivariate | Coefficient | P | Coefficient | P | Coefficient | P |
| Time (years) | -1.98 | 0.0978 | -8.1 | 0.0005 | -13.3 | P<0.0001 |
| Age† (years) | -2,782 | <0.0001 | 97.9 | <0.0001 | 5.12 | P<0.0001 |
| Adjusted R^2^ | 0.1732 | <0.0001 | 0.1922 | <0.0001 | 0.0629 | P<0.0001 |
|  |  |  |  |  |  |  |
| 2. COVID  (2020–2021) | n=761 |  | n=507 |  | n=2,267 |  |
| 2.1 Univariate | Coefficient | P | Coefficient | P | Coefficient | P |
| Time (years) | -79.0 | <0.0001 | 0.239 | 0.9902 | -49.1 | P=0.0038 |
| Adjusted R^2^ | 0.0273 | <0.0001 | -0.0020 | 0.9902 | 0.0033 | P=0.0038 |
| 2.2. Multivariate | Coefficient | P | Coefficient | P | Coefficient | P |
| Time (years) | -43.6 | 0.0025 | 15.8 | 0.3551 | -48.1 | P=0.0031 |
| Age (years) | -2,459 | <0.0001 | 98.2 | <0.0001 | 5.11 | P<0.0001 |
| Adjusted R^2^ | 0.2961 | <0.0001 | 0.2407 | <0.0001 | 0.0825 | P<0.0001 |
